# Plasmonic Nanobubbles as Tunable Cellular Probes for Cancer Theranostics

**DOI:** 10.3390/cancers3010802

**Published:** 2011-02-23

**Authors:** Dmitri Lapotko

**Affiliations:** Department of Physics & Astronomy, Department of Biochemistry and Cell Biology, Rice University, Houston, TX 77005, USA; E-Mail: dl5@rice.edu; Tel.: +1-713-348-3708, Fax: +1713-348-4150

**Keywords:** gold nanoparticle, laser, plasmonic nanobubble, theranostics

## Abstract

This review is focused on a novel cellular probe, the plasmonic nanobubble (PNB), which has the dynamically tunable and multiple functions of imaging, diagnosis, delivery, therapy and, ultimately, theranostics. The concept of theranostics was recently introduced in order to unite the clinically important stages of treatment, namely diagnosis, therapy and therapy guidance, into one single, rapid and highly accurate procedure. Cell level theranostics will have far-reaching implications for the treatment of cancer and other diseases at their earliest stages. PNBs were developed to support cell level theranostics as a new generation of on-demand tunable cellular probes. A PNB is a transient vapor nanobubble that is generated within nanoseconds around an overheated plasmonic nanoparticle with a short laser pulse. In the short term, we expect that PNB technology will be rapidly adaptable to clinical medicine, where the single cell resolution it provides will be critical for diagnosing incipient or residual disease and eliminating cancer cells, while leaving healthy cells intact. This review discusses mechanisms of plasmonic nanobubbles and their biomedical applications with the focus on cancer cell theranostics.

## 1. Introduction

Modern cancer research and treatment have several principal challenges. The first barrier in current clinical practice is the separation of diagnosis, therapy and therapy guidance into three independent stages. This both impedes treatment procedures and reduces their accuracy. Theranostics was recently introduced as a concept combining diagnosis and treatment [1-4]. Its successful realization will result in a significant improvement in both rapidity and precision in medical practice. The second limitation is the low sensitivity and specificity of current diagnostics and therapeutics that often cannot selectively identify and treat disease-specific cells and thus cannot support early-stage diagnosis and treatment. Being able to detect and treat disease selectively at cell level will result in earlier interventions and far higher treatment success rates. This requires a method and probe (agent) with tunable and multiple functions at the level of individual cells. The initial developmental phase of theranostics already revealed that such probes have not yet been developed [1-4]. Current probes do not provide true cell theranostics due to the limited tunability of the functions (diagnostic, therapeutic and guidance) of such agents as fluorescent and luminescent probes [2,5-11], micelles, polymers and liposomes [12-18], nanoparticles [2,6-8,19-24], plasmonic gold nanoparticles [2,3,25-35] and gas-filled and cavitation bubbles [36-49]. Despite widespread efforts, none of the above probes can be tuned in cells so as to support diagnosis, therapy and its guidance in a single process, with one probe, and at cell level. The specific function (diagnostic or therapeutic) of the nanoparticle is pre-set during its fabrication and cannot be changed after its delivery into the cell. So far the theranostic function was mainly achieved by combining several particles and molecules (each one supporting its specific function) into a single complex one. Examples of these are fluorescently labeled liposomes containing a drug, or micelles with a drug and a fluorescent label. Nanotechnologies were employed to address the above problems, but, despite the promise of the properties of nanoparticles, they still often use macroscale, rather than nanoscale processes and methods. This can be seen from the diagnostic and therapeutic applications of gold nanoparticles where the diagnosis is supported by optical scattering and the treatment is due to photothermally induced hyperthermia. Both phenomena high loads of nanoparticles in a target in order to provide diagnostic and therapeutic action, and thus their spatial scale is far bigger than “nano” [28-31,33,50-54]. This limits the accuracy, efficacy and safety of their medical applications.

**Figure 1.**
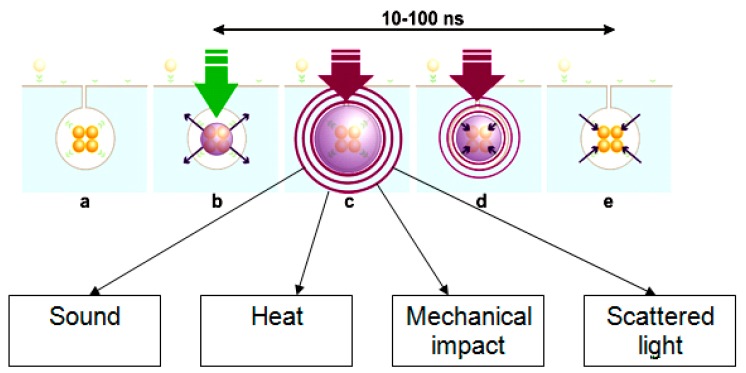
The mechanism of optical generation and detection of a plasmonic nanobubble: (**a**) the cluster of specifically targeted plasmonic nanoparticles (NPs) in the target cell; (**b**) a short optical excitation (pump) pulse is absorbed by the NP cluster and overheated NPs evaporate an adjacent layer of the cell volume thus creating a vapor nucleus around NPs; (**c**) the vapor expands into a nanobubble that scatters optical radiation of an additional, probe laser beam; (**d**) after reaching its maximal diameter the bubble collapses; (**e**) plasmonic NPs accumulate the heat that is generated during the collapse of a nannobubble and thus prevent the collapse-related effects on the NP environment.

We hypothesized that by combining the photothermal properties of plasmonic (gold) nanoparticles with the mechanical and optical properties of transient vapor nanobubbles we could produce a tunable theranostic probe. This probe is not a nanoparticle (NP) but an NP-generated event, a plasmonic nanobubble (PNB). A PNB (Figure 1) emerges when a plasmonic gold NP is locally overheated with a short laser pulse. As a result the NP evaporates a very thin volume (nanometer size) of the surrounding medium, thus creating a vapor nanobubble that expands and then collapses within a short nanosecond (Figure 1). Its fast expansion produces a localized mechanical and non-thermal impact. In addition, the bubble scatters the light, thus acting as an optical probe. We named this bubble as a plasmonic nanobubble because a plasmonic NP acts as its source and determines its parameters and location. Unlike other vapor bubbles (generated with bulk heating, ultrasound and lasers), a PNB thermally insulates the outer environment from the high temperature of a heated NP, thus reducing the risk of thermal damage to the minimum. The mechanical and optical properties of the bubble are determined by its maximal diameter (Figure 1) [55-57]. This, in turn, depends upon optical energy being absorbed and converted into heat by the plasmonic NP. Since the generation of a PNB occurs at the location of the NP, specific targeting of NPs will provide very specific and localized effects of PNBs. For these reasons, PNB properties can be remotely (activated with a laser pulse), precisely (at the location of NPs) and dynamically tuned with the energy of a laser pulse [55,56]. A PNB therefore acts as a tunable, multi-functional and on-demand agent, which does not exist until it has been activated with a laser pulse. The dynamic tunability of the properties of PNBs can support diagnosis, therapy and guidance of the therapeutic effect in individual cells in one connected and rapid theranostic procedure. Below we discuss the mechanisms of PNB generation, detection and tuning in individual living cells (Part 2) and their theranostic applications for cancer cells (Part 3).

## 2. Plasmonic Nanobubbles in Living Cells

### 2.1. The Mechanism of PNB Generation

The generation of a PNB (which is not a particle but a transient event) occurs through the optical excitation of plasmonic NPs with short laser pulses that causes NP heating and the consequent evaporation of the environment around the NP surface (Figure 1). The mechanism of generation of a plasmonic nanobubble includes several stages (Figure 1):

1. Absorption of optical energy by the NP through the mechanism of plasmon resonance causes the heating of the whole NP in 3–5 fs to a temperature that is above the evaporation threshold for the NP environment. The duration of the optical pulse must be short enough to concentrate the photothermal effect in a small volume around the NP.
2. Formation of a vapor nucleus around the NP occurs within 30–100 ps, but this vapor represents a layer of a nanometer thickness, and it is not a bubble since it cannot expand due to the limiting effect of the outer pressure (Figure 1b).
3. If the temperature of the NP is high enough, the pressure inside the vapor layer exceeds the outer pressure of the surface tension and the vapor begins to expand into a nanobubble. Such a nanobubble expands until the internal pressure reaches equilibrium with the outer pressure (Figure 1c); at this stage the diameter of the nanobubble is maximal. This process usually takes from several nanoseconds to microseconds and lasts longer than the excitation laser pulse. Therefore, the bubble expands due to previously accumulated energy and we assume that it is not supported by any energy source after the excitation laser pulse is terminated.
4. After reaching its maximal diameter, the bubble collapses back to the NPs (Figure 1d). The duration of the collapse stage is roughly the same as the expansion stage. The collapse of the bubble terminates its lifespan and includes an important role of plasmonic NPs: it accumulates the heat from the collapsing nanobubble and thus prevents extreme heating inside the nanobubble, the residual thermal impact to the environment after the collapse of the bubble (Figure 1e), and other events associated with the collapse of vapor bubbles, such as the generation of pressure and shock waves, plasma formation and sonoluminescence.

**Figure 2.**
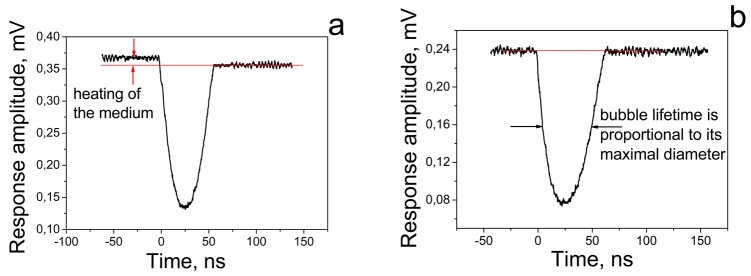
Time responses of the probe laser signal to single excitation laser pulses: (**a**) light-absorbing molecular solution generates the vapor bubble and residual heating of the environment (shown with two red arrows); (**b**) plasmonic (gold) NP generates the vapor bubble (PNB) without any environmental thermal effects after the collapse of the PNB. Red line shows the background level.

The mechanism of plasmonic nanobubble generation has several unique physical properties. Firstly, the location of the PNB is precisely determined by the location of the plasmonic NPs: a PNB will not emerge in an NP-free space, and this significantly improves the control of the PNB parameters (location, size and brightness) compared to any other laser-induced bubbles in cells and tissues. Secondly, the thermal impact of a PNB on the environment is minimal. This impact was experimentally analyzed through the time-responses of the laser-irradiated samples. The shape, amplitude and duration of optical time-response were earlier shown to directly characterize the phase state and the temperature elevation in a local volume that is exposed to the excitation laser pulse and is optically monitored with an additional probe laser [55,58]. We compared the time-responses of laser-heated homogeneous molecular solution and of gold NPs [57]. The photothermal effect in light-absorbing solution always included the two components, heating of the medium and bubble formation (Figure 2a). The two arrows in Figure 2a show the deviation of the post-bubble signal from the baseline due to residual heating of the surrounding medium. In the case of PNBs, no heating of the medium outside the bubble or after its collapse was observed (Figure 2b): the time response of the probe laser signal did not show any deviation from the background level. This difference can be explained by the insulating properties of the vapor around heated NPs: the low thermal conductivity of the vapor locks all the heat inside the bubble and thus prevents thermal impact on the environment. After the bubble collapse, the thermal impact is prevented by NPs that accumulate the additional thermal energy created during the bubble collapse. Therefore, PNBs differ in principle from both conventional photothermal bubbles and from plasmonic NPs. The latter are used for hyperthermia, as they convert optical energy and produce environmental temperatures below boiling point, thus delivering the heat into a significant amount of the surrounding volume. Only a PNB provides real concentration of the photothermal effect at nanoscale. As a result the impact of the PNB has a mechanical, non-thermal nature.

Thirdly, the generation of a PNB is a threshold phenomenon and occurs when the energy (fluence) of the excitation laser pulse exceeds a certain threshold. This threshold fluence was found to depend significantly upon the size and clusterization state of plasmonic NPs [55-57,59]. For single gold NPs, we found that an increase in the NP diameter from 10 nm to 80 nm (solid gold sphere) lowered the bubble generation threshold fluence by 18-times. Clusterization of the NPs showed a further decrease in the bubble generation fluence threshold. The plasmon properties of the cluster are different from those of an individual NP: the heating of several clustered NPs forms a joint vapor nucleus around them with the diameter being equivalent to that of a much bigger NP. This, in turn, reduces the optical energy (fluence) threshold required to form the PNB.

**Figure 3.**
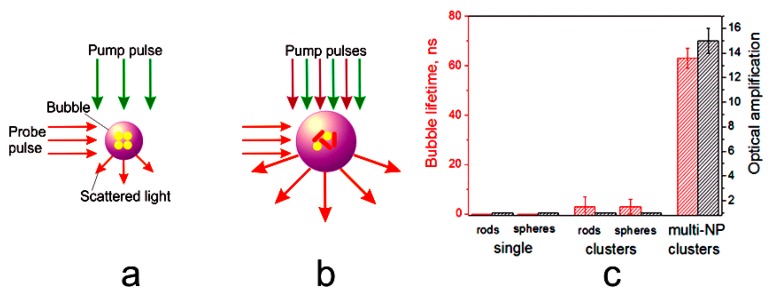
The mechanism of a rainbow PNB: (**a**) generation of the PNB with a single type of plasmonic NP and the matching excitation wavelength of a single laser pulse; (**b**) generation of the PNB with two types of plasmonic NPs (mixed in one cluster) and two simultaneous laser pulses having two different wavelengths that match each type of NP; (**c**) PNB parameters (lifetime and the relative amplification of optical scattering by a PNB compared to that of NPs) obtained under simultaneous excitation with two laser pulses at 532 nm and 787 nm for individual NPs and their clusters consisting of a single NP type (case (a)) and for the mixed cluster (case (b)) [60].

All of the above were considered for the excitation of a single plasmon resonance with a single optical wavelength that matched the peak of plasmon resonance of the NP. The targeting of specific cells with gold NPs often lacks the selectivity required for efficient diagnosis and therapy. We studied the synergistic effects of the simultaneous excitation of two plasmon resonances, visible and near-infra-red, in clusters of NPs consisting of two different types of NPs, spheres and shells. The NP clusters were activated optically with short laser pulses so as to achieve the selective generation of plasmonic nanobubbles around the clusters with mixed NPs. This method, referred to as rainbow nanobubbles [60,61], was recently developed and applied to the selective targeting and activation of plasmonic nanoparticles in prostate cancer cells mixed with non-cancer cells. The NP cluster consisting of the two types of NPs with different excitation wavelengths, was exposed to a pair of simultaneous optical pulses at two different wavelengths that matched each plasmon resonance. The fluence of each pulse was below the PNB generation threshold for the clusters consisting of the single type of NP (Figure 3), and only synergetic and simultaneous absorption of all laser pulses by the multi-NP cluster exceeded the PNB generation threshold. This result was obtained for the clusters of gold spheres, nanorods and the mixed clusters of spheres and rods (Figure 3). Simultaneous laser pulses generated PNBs only in the mixed NP clusters and amplified optical scattering brightness by 12–18 times, thus demonstrating both specificity and sensitivity. We refer to this mechanism as “rainbow plasmonic nanobubbles” [60,61].

The mechanism of PNB generation is based on the interaction of short optical pulses and plasmonic NPs. Plasmonic NPs are known for their outstanding photothermal [29,32,50,62-64] and optical scattering [27,28,31,62,65-67] properties. Short optical pulses and high-energy excitation of plasmonic NPs create an “NP-vapor-liquid” system, whose thermal, mechanical and optical properties principally differ only from those of the plasmonic NP. Vapor bubbles can be generated in a medium with a laser pulse in the absence of a plasmonic NP through several other mechanisms: optical breakdown [39,40,42,68], photothermal effect and ultrasound [38,69-74]. Although laser-induced macro- and micro-bubbles were extensively studied [38-41,75] and the optical properties of acoustically and optically generated bubbles are well recognized [69,70], the mechanisms of bubble generation (optical molecular absorbance on a microscale, rarefaction and optical breakdown), do not provide for the precise control of the bubble parameters at nanoscale. This lack of control over the bubble parameters may limit their applications where the mechanical, thermal and optical impacts of the bubble require precise tuning and control at nanoscale. Although the femtosecond laser microsurgery methods have a high precision of mechanical impact [40,42,76], such methods also require very precise positioning of the laser beam waist relative to the target and this limits the application of the femtosecond methods. The main difference between PNBs and heated plasmonic NPs can be summarized as being the threshold and tunable nature of the thermal, mechanical and optical effects of a PNB and as its transient nature (Table 1).

Experimental studies of bubble generation around plasmonic NPs include several reports, some of them using interesting methods [32,77-83]. However, these studies have two limitations: either the bubbles were not detected directly, or the individual NPs were not studied, both of which limit the accuracy of the data and the conclusions. Furthermore, NP-generated bubbles were not directly detected in individual living cells and tissues. To distinguish the origin and the scale of plasmonic NP-generated bubbles from nanoparticles and from vapor bubbles of another origin, we define them as plasmonic nanobubbles. The new technologies and methods which we have developed recently and described below, allow the precise generation and direct imaging and monitoring of the NP-generated PNBs *in vitro* and *in vivo*.

### 2.2. Cell Targeting with Plasmonic Nanoparticles for PNB Generation

**Figure 4.**
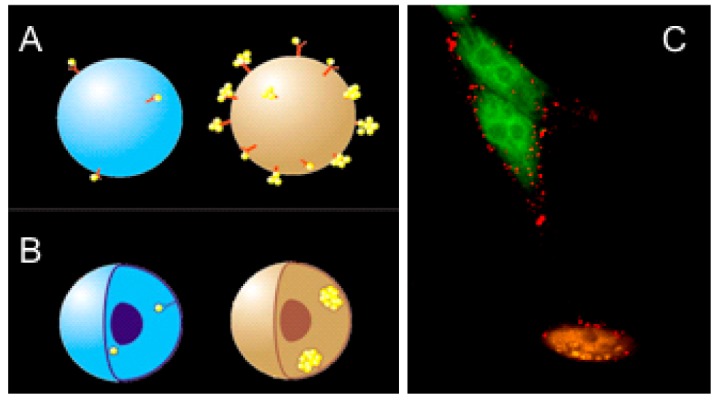
The mechanism of NP targeting ((**a**) gold conjugates with cell-specific antibodies target the matching receptors, (**b**) membrane-located NPs are endocytosed and selectively form NP clusters in cells with sufficient amounts of NPs) and laser confocal scattering and fluorescent images of the target (green) and normal (amber) cells, (**c**) optical scattering by gold NPs is shown in red: Only the target cell (green (treated with AlexaFluor 488)) built several large NP clusters while the normal cell (amber (treated with AlexaFluor 546)) non-specifically accumulated some NPs without creating large NP clusters.

**Figure 5.**
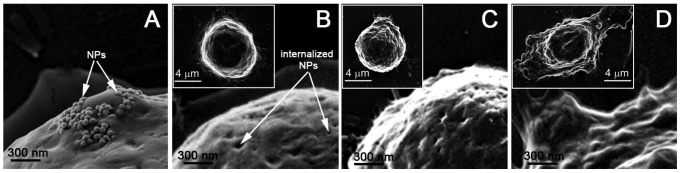
Scanning electron microscopy images of cancer cells after incubation with gold NPs show their membrane coupling (**a**) and internalization (**b**), and the result of the generation of a non-invasive PNB with a lifetime of 25 ± 5 ns (**c**) and an ablative PNB with a lifetime of 300 ± 42 ns (**d**) [96]. The insets show images of the whole cells. The cells were fixed immediately after being treated with PNBs.

As we described above, a PNB is not a particle but a stealth agent (event), activated on demand with an excitation (pump) laser pulse around a single or clustered plasmonic NPs. Therefore, the generation of a PNB requires the efficient targeting of plasmonic NPs to specific cells. The purpose of NP delivery to the cells is to provide the formation of target-specific NP clusters to minimize the influence of the non-specific accumulation of NPs. This is achieved in two steps:

Firstly, the NPs are covalently conjugated to the disease-specific antibody that attaches the NPs to the matching receptors at the surface of the cellular membrane during their incubation with the NP conjugates. This provides the accumulation of the NPs in a cell but shows limited selectivity due to the non-specific coupling of the NPs to normal cells or to cells with low levels of the expression of similar receptors (Figure 4a and 4c, Figure 5a).

Secondly, we use receptor-mediated endocytosis to aggregate NPs into the clusters (Figure 4b and 4c), Figure 5b) formed with endogenous ligands [84,85]. Clusterization of NPs was shown to decrease the PNB generation threshold fluence of laser radiation [55-57,59,84-86], and thus NP clusters allow the generation of PNBs around them at the relatively low fluence levels that exclude the generation of PNBs around single NPs or around their small clusters.

NP safety is the subject of massive studies and was reviewed in [87-89]. Among all NPs the gold ones were found to be the safest [87] and they are also FDA-approved. We experimentally monitored the viability of cells after their interaction with gold spherical NPs by using propidium iodide (PI) dye (which penetrates through the damaged membrane and thus stains the dead cells) and flow cytometry. Incubation at 4 °C did not have any considerable negative effect on the cells. For K562 cells, the level of PI+ cells changed from 2% before targeting to 2.8% after targeting. Incubation at 37 °C resulted in additional damage to the cells, increasing the level of PI-positive cells up to 3.4%. Nevertheless, these values indicate that the targeting of cells with NP and the processes of NP clusterization are relatively safe. This is partly due to the non-toxic nature of gold NPs that were shown to have negligible toxicity on living cells [87-89]. The viability of the cells after their incubation with gold spheres (NSP) and gold nanorods (NR) was measured with a Trypan Blue exclusion test. The initial viability of the cells prior to their incubation with NRs was 95% ± 3%. After incubation with NR-C225 for 30 minutes, the viability of the cells was found to be in the range of 80% ± 10%. The viability of the control intact cells also slightly decreased to 89% ± 4%. The conjugates of NR-C225 were not absolutely safe for the cells. Their toxicity could be caused by minor amounts of residual CTAB molecules that might be left on NRs after their conjugation with MAB. Currently, there are opposing points of view on the cytotoxicity of NR with CTAB: some groups have reported that CTAB does not influence the viability of cells [90-92], while others found a strong cytotoxic effect of CTAB [93-95]. We consider that CTAB-associated NRs are toxic by definition. In addition, CTAB coated NRs are internalized by cells via a nonspecific uptake mechanism [34,91] and thus the selectivity of cell targeting is reduced.

Therefore we may conclude that the interaction of antibody-functionalized NPs with living cells selectively creates clusters of many closely located NPs that are linked through specific antibodies and/or by cellular structures such as endosomes and vacuoles. The advantages of clusters instead of single NPs for medical diagnostics and therapy using optical methods include much greater volume, which allows effective photothermal interactions under conditions of relatively slow heat diffusion. The formation of NP clusters does not compromise the viability of the cells. Additional details can be found in [59,84,97-100].

The concentration of NPs during cell targeting and the actual amount of internalized NPs are important parameters related to the efficacy of PNB generation, administration of NPs and their safety. While the targeting concentration of gold NPs in most of the studies varied in the range of 5 × 10^9^−1.3 × 10^10^ particles/mL, the internal concentration requires an additional definition. For PNB-based diagnosis and therapy it is sufficient to generate at least one PNB per cell. This means that one big cluster of 30–60 NPs will support diagnostic and therapeutic functions. Providing heterogeneity of NP internalization and clustering in living cells, we may estimate the required load to be 300–600 NPs per cell. An electron microscopy-based direct count of 30 nm gold spheres in leukemia cells [84,99] confirmed that the cell volume-averaged concentration of gold NPs increased (relative to the targeting concentration) by one order of magnitude (10^11^ ± 4.7 × 10^11^), while in the areas with NP clusters it was much higher, up to 10^13^ NP/cm^3^. These estimates have to be correctly understood for the cluster-threshold mechanism of PNBs: even 100 NPs per cell when aggregated in at least one big cluster may support all PNB functions.

Compared to other methods that employ gold NPs for diagnosis (optical scattering [28,50-52] and photoacoustic imaging [101-103]) and for therapy through hypothermia [29,30,33,50,51,53,54] and drug delivery [104-107], we point out that the cluster-threshold mechanism of PNB generation and detection tremendously reduces the actual load of NPs required for detecting the target cell and for its destruction (Table 2).

### 2.3. Optical Detection of PNBs

**Figure 6.**
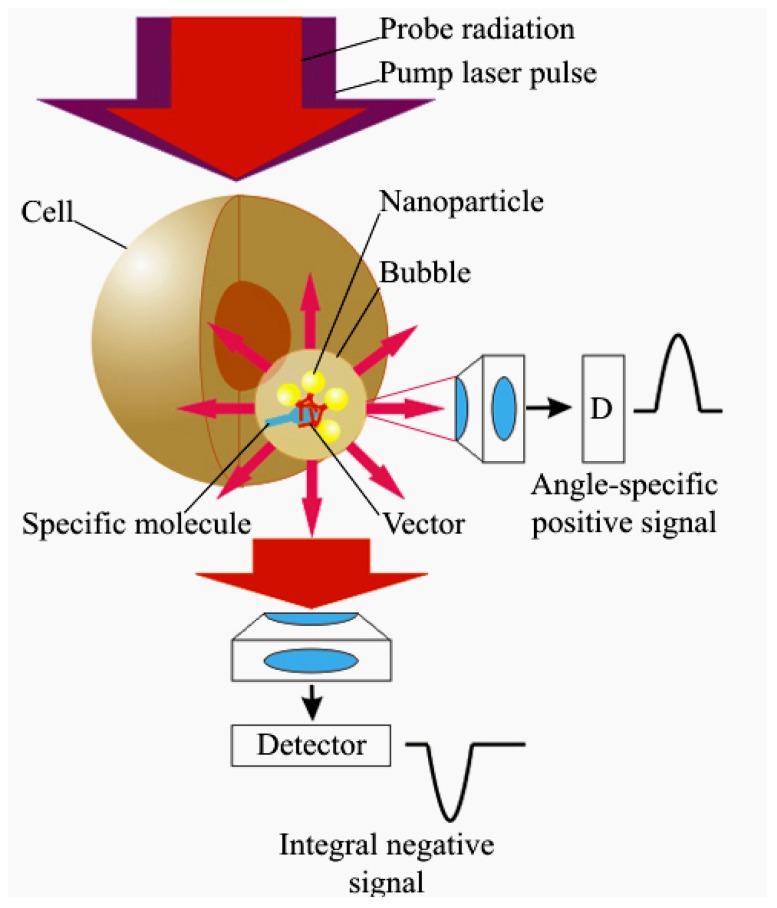
Principle of PNB amplification of optical scattering from an intracellular target: amplitude of the optical signal can be increased through formation of a target-linked NP cluster, and through generating a transient bubble around the NP cluster; the PNB may be detected with a scattered probe laser beam (pulsed or continuous) as an angle-specific positive signal or an integral negative signal [96].

The generation and impact of PNBs in individual living cells require remote and efficient monitoring. PNBs can be detected optically since the vapor-liquid boundary (PNB surface) creates strong scattering of light. The brightness of the scattered light rapidly increases with the size of the scattering object, and in the case of PNBs is determined by their maximal diameter. In other words, the optical properties of a PNB directly depend upon its thermo-mechanical properties, since the maximal diameter depends upon the energy of the excitation laser pulses. The most important optical property of a PNB is its ability to amplify such scattering, compared to that of gold NPs (which are used to generate the PNB). The optical amplification effect of the PNB is due to its increased diameter and to the high gradient of the refractive index that emerges at the bubble-environment boundary. Increased brightness and stability of the PNB as an optical probe is achieved by using optical scattering instead of optical emission (Figure 6). Neither a PNB nor an NP emit light, instead the PNB scatters incident probe optical laser radiation. The wavelength of the probe laser is chosen in a range that allows significant optical energy of the probe laser without the risk of photodamage to biological objects and to the NPs. Since the energy scattered by a PNB depends upon the incident energy, and since no emission process is involved, the brightness of the PNB can be increased without the risk of photodamage.

**Figure 7.**
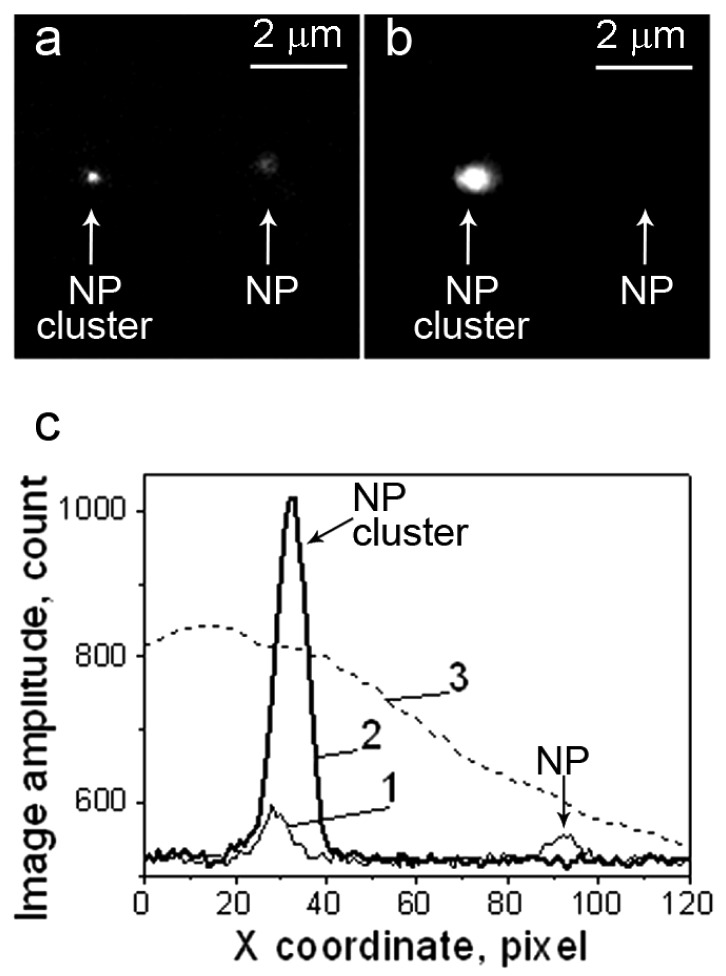
(**a**) optical scattering images of a 90 nm gold NP and a cluster of 90 nm NPs; (**b**) the same objects showing attenuation and amplification of scattering upon their exposure to a pump pulse (532 nm, 0.5 ns) that induced a bubble around the NP cluster but not around the NP; (**c**) the spatial profile of the pump laser (3) shows the difference in the fluence applied and the profiles of the scattering amplitudes: 1—case (a), 2—case (b) [56].

Depending upon the size of the bubble, it will attenuate or will amplify optical scattering. We recently verified this property of PNBs in experiments with gold NPs and their clusters in water [56,57] and in living cancer cells [85]. The cluster mechanism of PNB generation demonstrates the dependence of PNB brightness upon the NP clusterization state (Figure 7). Compared to gold NPs at room temperature (cold), we found that at high laser-induced temperatures, the properties of “hot” gold NPs are mainly determined by the properties of the two-phase (vapor-liquid) environment, in other words, by the properties of PNBs. As a result, the optical properties of the nanoparticle-bubble system are strongly influenced by the bubble generation: the optical attenuation around single NPs in Figure 7b, and the optical amplification by 12-times around an NP cluster (Figure 7b). The bubble also improves the optical contrast of NP clusters compared to single NPs by 27-times [56].

**Figure 8.**
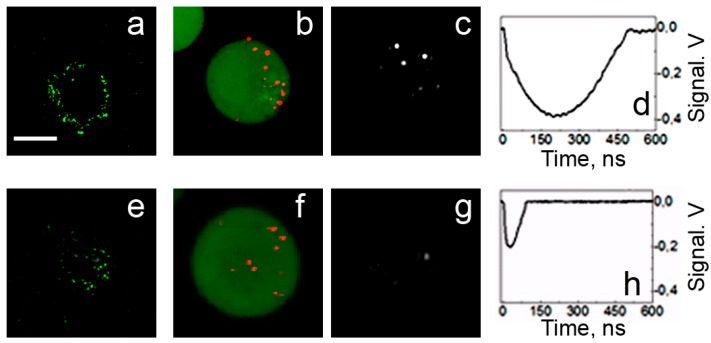
Confocal fluorescent, confocal scattering and time-resolved scattering images of prostate cancer C4-2B (**a**,**b**,**c**) and stromal HS-5 (**e**,**f**,**g**) cells: (**a**,**e**)—confocal fluorescent images of AlexaFluor488 conjugated to PSMA antibody [60], (**b**,**f**)—confocal scattering images of gold NPs (shown in red on a green fluorescent background that shows cell tracker dye), (**c**,**g**)—time-resolved scattering images of the cells being exposed to a simultaneous pair of laser pulses at 532 nm and 787 nm and (**d**,**h**)—the corresponding time-responses of PNBs [61]. Scale bar is 10 μm.

Optical scattering can be detected from zero level when the light scattered by a PNB is registered in a specific direction, thus producing a positive signal (Figure 6). Alternatively, the probe beam can be directed into the sensor so that the scattering in all directions by the PNB decreases the amount of light at the detector, thus producing a negative signal (relative to the initial baseline) that characterizes an integral scattering effect (Figure 6). Besides the pulsed probe beam, the continuous illumination and detection can be used to register the kinetic behavior of the scattering signal. We defined the continuous optical monitoring of the PNB as a response mode and the time-resolved pulsed imaging of the PNB as an image mode. Image and response modes are used simultaneously thus combining PNB imaging and measurement of its lifetime. The sensitivity and specificity of PNBs as optical cellular probes was evaluated by comparing PNB brightness and lifetime for cancer *versus* normal cells. Prostate cancer C4-2B cells and non-cancerous HS-5 stromal cells were imaged with fluorescence of AlexaFluor488 marker (targeted with prostate cancer-specific PSMA antibody), scattering by gold NPs (targeted with the same antibody) and scattering of the PNBs generated around gold NPs (Figure 8). PNBs were detected in individual living cells as two simultaneous optical signals: a time-resolved optical scattering image with a pulsed probe laser (Figure 8c and 8g) and a time-response that showed the dynamics of the growth and collapse of the PNB (Figure 8d and 8h). The brightness of each image was quantified as a pixel amplitude and the cell population-averaged values for cancer cells were divided by the corresponding values obtained for non-cancerous cells. Thus we obtained optical contrasts for fluorescent imaging (1.9), NP scattering (1.5) and PNB imaging (5.8) [60,61].

The brightness of the PNBs in cancer C4-2B cells (measured as the pixel image amplitude of the PNB, Figure 8c) was found to be 71-times higher than that for stromal cells (Figure 8g). Such an optical contrast exceeded the optical contrast of the fluorescent labels (that were targeted to C4-2B and HS-5 cells using the same prostate cancer-specific PSMA antibody, see Figure 8a an 8e) by 31-times. The optical contrast (measured as a ratio of the fluorescent image amplitudes for C4-2B to HS-5) of fluorescent imaging for cancer *versus* stromal cells was 2.3 (Figure 8a and 8e). The accumulation of the gold NP conjugates in cells was imaged by using the optical scattering mode of the laser scanning confocal microscope (Figure 8b and 8f). Such a high contrast of PNB imaging was provided by the threshold mechanism of PNB generation: no or only small PNBs were generated in HS-5 cells because the level of laser fluence was close to the PNB threshold for the smallest clusters formed in HS-5 cells, while the same fluence level exceeded the PNB generation threshold for significantly larger clusters formed in C4-2B cells [60,61].

**Figure 9.**
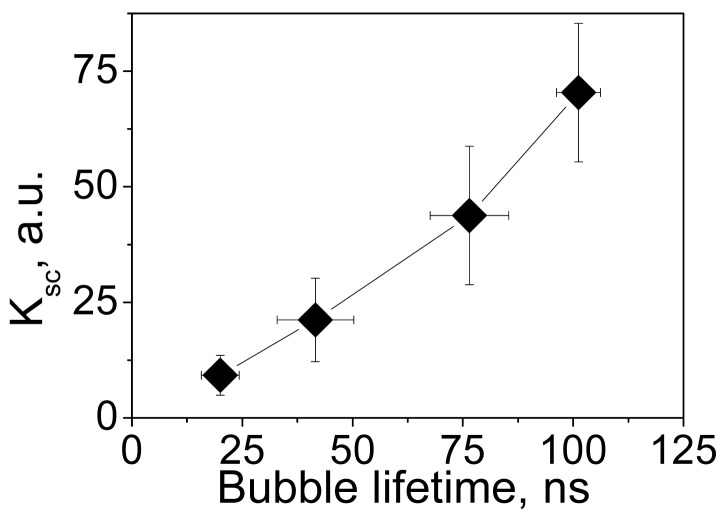
Influence of the fluence of a single pump laser pulse (532 nm, 0.5 ns) on the amplification of optical scattering signal by a PNB (relative to gold NPs) in NP-treated cells as a function of PNB lifetime (*i.e.*, maximal size of the PNB) [85].

The brightness of a PNB can be dynamically tuned with an excitation laser pulse through its fluence level (see p. 1.5 below). For a quantitative analysis of the optical amplification by PNBs relative to optical scattering by gold NPs, we have introduced the relative scattering image amplitude *K_sc_(t)* = *[I(t)* − *I_b_]*/*[I(0)* − *I_b_]* that describes the pixel image amplitude *I(t)* of optical scattering by a PNB, relative to that by an NP *I(0)* (*I_b_* is the average pixel image amplitude of the background). Figure 9 shows the dependence of the optical amplification of PNB optical scattering upon the maximal size of PNB that is controlled through the laser fluence.

### 2.4. PNB and Cell Damage

**Figure 10.**
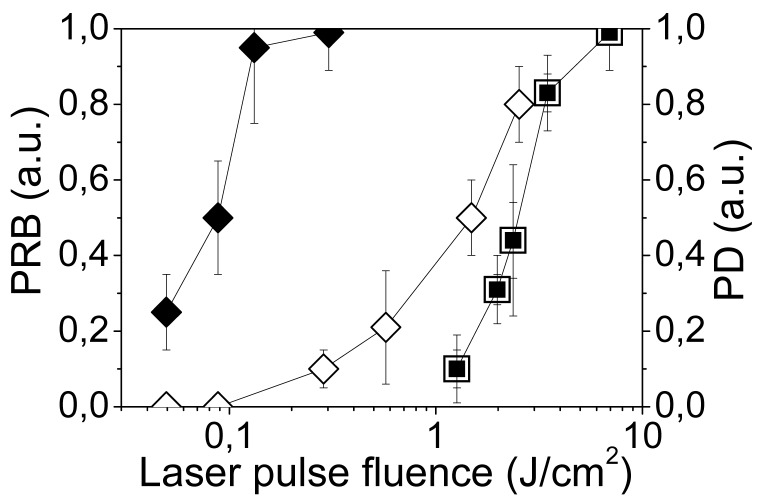
Influence of the fluence of a single pump laser pulse (532 nm, 0.5 ns) on the PNB generation probability (PRB) and on the damage as measured in individual A549 cells: ◆—cells incubated with NP-C225 conjugates, ■—intact cells; cell damage probability (PD): ◊—cells incubated with NP-C225 conjugates, □—intact cells [85].

We studied the cell damaging properties of PNB's by varying the laser pulse fluence in order to analyze the probability of bubble generation and the probability of cell damage among intact (untreated) and NP-treated cells. Cell viability was evaluated optically with two standard microscopy techniques. Firstly, a bright field image was obtained for the cell before and after its exposure to a single pump pulse and the difference between these two images was used to detect any PNB-induced changes in the shape of the cell, in particular, the emergence of blebbing bodies. Blebbing bodies may develop in cells with a damaged cytoskeleton and even with an intact membrane. Secondly, membrane damage by the PNB was detected using a standard fluorescent method by monitoring the cellular uptake of Ethidium Bromide (EtBr) dye that only enters the cells with a compromised membrane. Fluorescent images were obtained for each cell before and after PNB generation. Though these methods did not provide monitoring of long term viability, they could be applied on site and to specific individual cells during PNB generation, and without removing the cells from the sample chamber. Each single cell in the population was irradiated with a single laser pulse of a specific fluence, and then the cell population-averaged values were obtained (Figure 10). NP treatment lowered the threshold laser fluence for bubble generation by almost 30-times relative to the intact cells (Figure 10, Table 3). As a function of pulse laser fluence, the probabilities of cell damage and of bubble generation coincided for intact cells, but were significantly separated in the NP-treated cells (Figure 10). At pulse fluences of 0.1–0.4 J/cm^2^, intracellular PNBs were generated in NP-treated cells without damaging the majority of the host cells (Figure 10), while the same cells were damaged at 10-times higher fluences (Table 3).

We simultaneously measured the lifetime of the PNB in each irradiated cell as a function of the laser pulse fluence. The lifetime of the non-invasive PNBs was found to be about 5-times shorter than that of the damaging ones (Table 3). This implies a similar difference in the maximal diameters of non-invasive and damaging PNBs. We consider that the maximal diameter of a PNB plays a major role in cell damage: small PNBs do not damage the cell, while an increase in PNB size by several times induces almost immediate disruption of the cellular membrane and skeleton as was revealed with electron scanning microscopy.

Based on the results obtained, we estimated the cell damage threshold lifetime of a PNB to be about 110 ns. We found that intact cells cannot support such small non-lethal PNBs (Table 3) and that the generation of photothermal laser-induced bubbles was always associated with cell damage [108-111], suggesting that the endogenous optical absorbers in intact cells cannot generate small PNBs. We also found that at laser fluences below the PNB generation threshold, the NPs in cells were still significantly heated by the laser pulse, but did not cause detectable damage to the cells. Also, the exposure of the cell to 16 pump laser pulses (at 15 Hz frequency) instead of a single pulse did not influence the cell viability and the level of the damage threshold fluence, which suggests that cell damage results from a single event rather than from the accumulative effect of a sequence of PNBs. Thus, the PNB damage mechanism is mechanical, non-thermal and rapid: a single laser pulse induces an expanding PNB that disrupts the cellular cytoskeleton and plasma membrane causing blebbing and cell staining with the membrane-penetrating dye.

**Figure 11.**
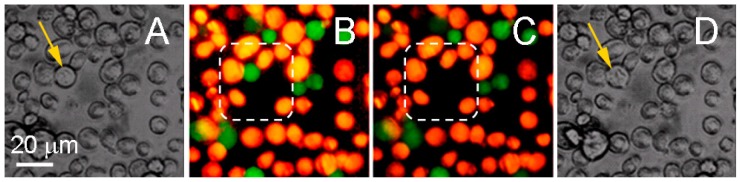
Bright field image and calceins fluorescence of prostate cancer C4-2B (green) and stromal HS-5 (orange) cells before (**a**,**b**) and 60 s after (**c**,**d**) exposure to a pair of laser pulses (532 nm and 787 nm) that selectively generated a cell damaging PNB causing fading of green fluorescence due to leaking of green calcein out through the disrupted membrane. Dashed line shows the area that was exposed to the laser pulses. Bright field images show the disruption of the target cell [61].

We evaluated the selectivity of the PNB-induced damage of cancer cells when surrounded by non-cancer stromal cells expected to be in the typical cancer microenvironment. We fluorescently labeled stromal (HS-5) and prostate cancer cells (C4-2B) with calcein vital dyes of green (cancer) and orange (stromal) colors and mixed them in the proportion of HS-5:C4-2B as 5:1. We treated the mixture of the cells with two gold conjugates using PSMA (prostate cancer specific) and C225 (anti-epidermal growth factor receptor) antibodies. Large PNBs were generated only in cancer cells. PNBs were also observed on occasion in stromal cells, but the probability was below 20% and their size was small compared to the size of cancer cell-generated PNBs. Within the area irradiated with laser pulses (shown within a dashed line in Figure 11) we discovered that only the cancer cell was damaged [61]. The vital dye stays in the cell with an intact membrane and quickly leaks out of the cell with a compromised membrane [112]. The damage to the cancer cell in the center (observed as the loss of green fluorescence) was in line with the increased size (lifetime) of the PNBs generated specifically in C4-2B cells (see Figure 11), and with the mechanical nature of cell damage that is associated with the disruption of the cellular structures including the plasma membrane [58,85,96,113]. This experiment clearly demonstrated the selectivity of PNBs.

### 2.5. Dynamic Tunability of PNB Size, Brightness and PNB Generation Threshold

**Figure 12.**
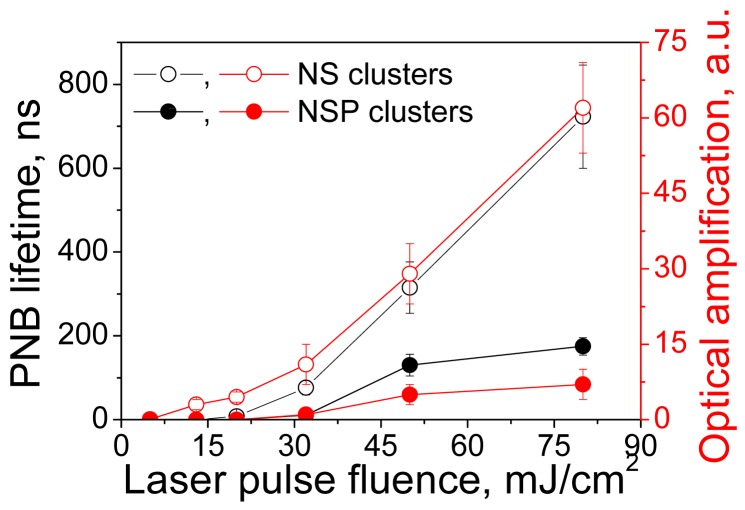
Influence of the fluence of a single pump laser pulse (532 nm, 0.5 ns) on the PNB lifetime and brightness (the latter is show as amplification of optical scattering signal by a PNB relative to scattering by NP cluster) for: clusters of gold hollow nanoshells (NS)—hollow dots, clusters of gold spheres (NSP)—solid dots.

The ability to dynamically (*i.e.*, in real-time) tune the mechanical impact and brightness of the cellular probe are the most promising features of a PNB. Physically, the levels of mechanical impact and of brightness are determined by the maximal diameter of the PNB. This can be directly measured for individual PNBs through their lifetimes, that correlate with the PNB maximal diameter [41,42,114,115]. Optical scattering and mechanical properties of the plasmonic nanobubble above the generation threshold can be tuned through the fluence of the pump laser pulse thus providing precise control of plasmonic nanobubbles. We simultaneously measured the lifetime and brightness of individual PNBs generated around the clusters of hollow nanoshells (NS) and of solid spheres (NSP) of identical size (around 50-60 nm) as a function of the laser pulse fluence (Figure 12). For relatively small PNBs their lifetime was found to be nearly proportional to their maximal diameter [41,42,114,115] and was used as the measure of mechanical impact of PNB that is determined by its maximal diameter. PNB lifetime increased linearly with the laser fluence for PNBs generated around both types of NP clusters after the excitation fluence of the laser pulse exceeded the PNB generation threshold level. The optical scattering brightness of these PNBs also increased with the laser fluence and correlated to their lifetimes (Figure 12). However, the thresholds for shells and spheres differed significantly despite their almost identical sizes. It should be noted that the PNBs were generated in both cases under the fluences and optical doses that were well below the FDA/NTSI safety thresholds and also much lower than the doses associated with another gold NP-based methods (see Table 6 for detailed comparison of optical doses). Shells were found to be much more efficient for the generation of PNBs and this is related to their reduced thermal capacity. The details of the physical mechanisms of PNB generation can be found in [55,57]. In all cases the PNB size was precisely controlled by the fluence of the excitation (pump) laser pulse.

The generation of a PNB has a threshold nature, a PNB cannot emerge around an NP if the excitation fluence is below a specific level. The NP cluster-threshold mechanism makes the generation of PNBs very selective, as can be seen from Figure 12. The PNB generation threshold fluence was found to be determined by nanoparticle size, heat capacity, aggregation state, and the pump laser pulse duration [55-57,99,116-118]. Low PNB generation threshold fluence can be achieved with NPs with a high ratio of surface to heat capacity (such as in nanoshells), through NP clusterization and through the shortening of the laser pulse to a level below the characteristic time for thermal relaxation of the NPs or their cluster. The generation of a PNB does not coincide with the formation of vapor around the surface of a transiently heated plasmonic NP, and requires additional energy that significantly exceeds the explosive boiling threshold. This additional and higher threshold also results in the limitation of the minimal lifetime (and hence maximal diameter) of PNBs at the level of 9 ns [57]. A possible explanation for the discovered mechanism of PNB generation relates to the effect of surface tension pressure that is applied to the vapor nucleus near the NP surface. Due to the small size of NPs, the surface tension pressure around the NP (and around the vapor nucleus forming around the NP) may be very high, thus preventing PNB expansion (as can be expected for macro-bubbles). As a result, additional optical energy is required to generate a PNB.

**Figure 13.**
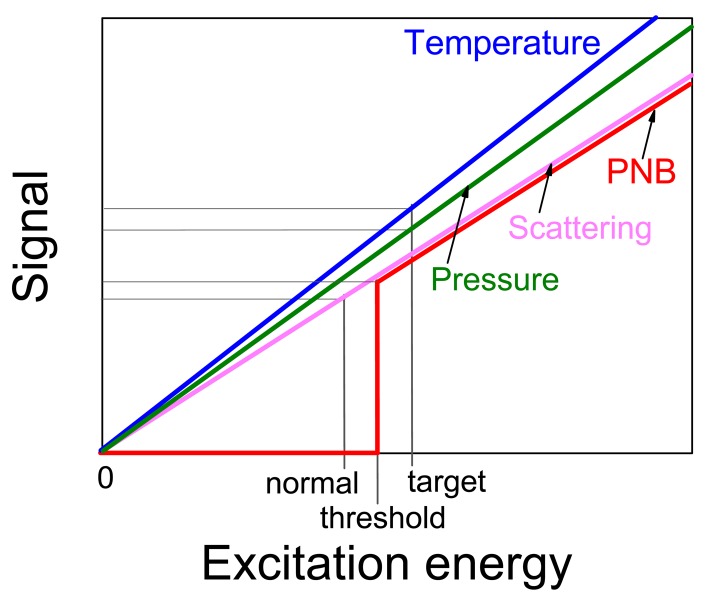
Signals from the two different cell, normal cell (NP cluster-negative) and target cell (NP cluster-positive) generated by several non-threshold (thermal, acoustical, optical) and threshold (PNB) optical processes as function of the excitation energy. In case of the threshold process the normal cell returns zero signal (no bubble) thus providing maximal contrast and selectivity relative to a target cell.

In terms of distinguishing the target and non-specific cells the threshold mechanism of PNB generation increases the contrast and specificity of their detection: assuming that the NP clusters in target cells will be larger than those in non-specific cells we may adjust the excitation (pump) fluence to the level that exceeds the PNB threshold for target cells though is below much higher PNB threshold for non-specific cells. In this case PNB s will be generated only in target cells. Another methods use non-threshold processes for cell imaging (photothermal, photoacoustic, fluorescent, scattering) and, therefore, provide only incremental difference in the signals between target and non-specific cells with much lower specificity compared to qualitative difference between PNB-positive and PNB-negative cells (Figure 13).

**Figure 14.**
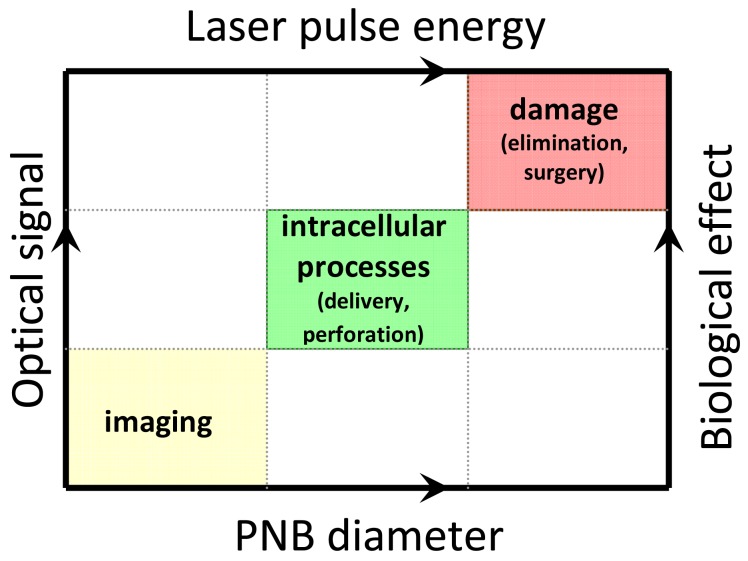
Tunability and guidance of the biological effects of PNBs: PNB diameter determines specific biological action and optical signal, while the pump pulse parameters determine the PNB diameter.

The dependence of the diagnostic (optical) and therapeutic (mechanical) effects of a plasmonic nanobubble upon its diameter creates a unique opportunity for real-time (dynamic) tuning and remote monitoring of the biological effect of a PNB in an individual cell *in vitro* and *in vivo* by varying the PNB diameter through the pump laser pulse fluence (Figure 14). We suggest that PNBs with a diameter smaller than 300 nm are nonlethal to living cells and are applicable to non-invasive imaging. Those in the range of 400 -1000 nm produce local reversible mechanical impact and be applicable to delivery and other manipulation at sub-cellular level without irreversible damage to the cell. PNBs larger than 1 μm mechanically and irreversible damage the cell and can support therapeutic applications. We consider the dynamic tunability of the PNB as the central strategy for new biomedical applications that include theranostics as well as nano-and micro-surgery [119,120] and intracellular targeting [119].

## 3. PNB theranostics

Combining diagnosis and therapy in one process is an emerging biomedical method referred to as theranostics [4,121]. A distinct goal of theranostics is to selectively target specific (diseased) tissues or cells to increase diagnostic and therapeutic accuracy. The major promise of theranostics is to bring together the key stages of a medical treatment such as diagnosis and therapy, and thus to make treatment shorter, safer and more efficient. We hypothesized that a combination of the photothermal properties of plasmonic nanoparticles with those of transient vapor bubbles may be the key to solving the above problems by allowing us to develop a tunable nanoscale theranostic probe.

### 3.1. Principle of PNB Theranostics

**Figure 15.**
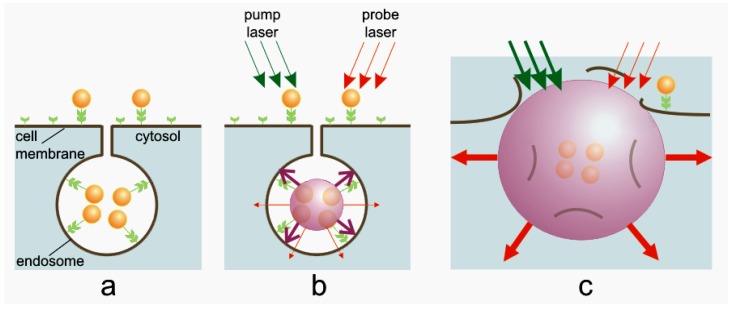
PNB cell theranostics with a multi-stage tunable PNB: (**a**) the cell is targeted with NP-antibody conjugates and intracellular NP clusters are formed through receptor-mediated endocytosis, (**b**) the 1st (diagnostic) PNB provides the data on the cell and allows us to determine the parameters of the next laser pulse, (**c**) the 2nd PNB delivers mechanical impact (cell damage though membrane disruption is shown) and this action is guided through the increased optical scattering (red arrows) of the 2nd PNB; the PNB is tuned by varying the fluence of pump pulse (green arrows) [85].

For the target-specific generation of PNBs we selectively formed clusters of relatively safe gold NPs around molecular targets in cancer cells. Gold NPs, conjugated to diagnosis-specific antibodies were delivered and aggregated into NP clusters through the mechanisms of antibody-antigen interaction and endocytosis (Figure 15a). Remote (optical) and non-invasive activation and sensing of PNBs around such intra-cellular clusters was realized in individual living cells with free laser beams. When activated by a laser pulse, an intracellular plasmonic nanoparticle (Figure 15b) acts a heat source and generates a transient PNB in the surrounding medium. PNBs of nanometer-scale size and nanosecond-scale duration act as diagnostic probes by scattering the light from a probe laser. Larger micrometer-scale PNBs provide a localized therapeutic action through a mechanical (non-thermal) impact due to their rapid expansion and collapse, thus disrupting the cell membrane (Figure 15c). Optical monitoring of the disruptive PNB's can guide their therapeutic action. Thus PNB's may combine diagnostics, therapy, and therapy guidance.

**Figure 16.**
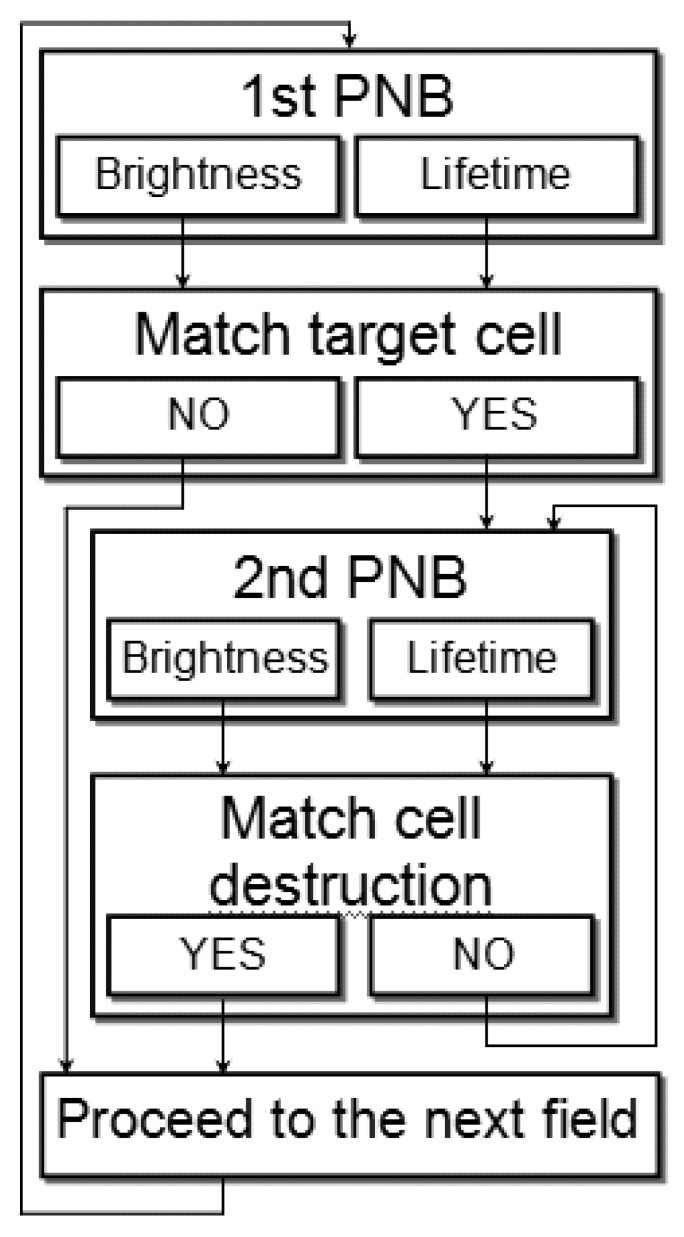
Algorithm of cell theranostics with two sequential PNBs of different sizes provides the three connected steps of diagnosis, cell ablation and immediate guidance of the cell ablation through PNB optical scattering.

The theranostic algorithm in individual cells was designed in order to discriminate between normal and diseased cells. We describe an automated algorithm that combines diagnostic and ablative modes through immediate feedback to detect and kill a target cell. This algorithm (Figure 16) probes the cells with a range of laser fluences and employs two sequential pulses: the first PNB detects the cell, and if its lifetime (or brightness) match the window for the target cell, the second PNB of increased diameter is rapidly generated after the first one for the therapeutic ablation by immediately increasing the fluence of the laser pulse above the cell damage threshold (Figure 16). The threshold fluence of PNB generation was found to be lower than the biological damage threshold fluence [85,96]. Therefore, by varying the fluence of the laser pulse from sub-lethal to lethal levels, we can efficiently switch from a non-invasive to an ablative mode.

The speed of this process is limited by the speed of pulse generation and can reach 10 μs for the whole algorithm. Such speed will allow high throughput for *in vivo* applications enabling efficient scanning of the area (or volume) that need to be treated.

### 3.2. Cell Theranostics with PNBs: in vitro Studies

**Figure 17.**
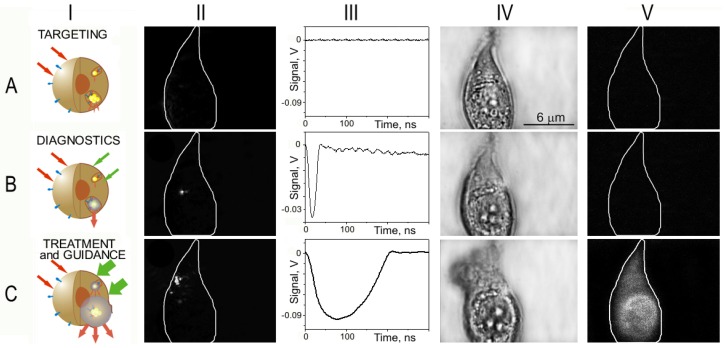
Targeting the cell with gold NPs (**a**) and the optical generation and detection of intracellular PNBs: the 1st PNB non-invasively amplifies optical scattering (**b**), while increasing the fluence of the pump laser pulse induces the 2nd PNB that mechanically damages the cell (**c**); I—stages of PNB theranostic action; II—optical pulsed scattering images of one cell with the membrane border shown with a white line; III—optical time response of the PNB shows its lifetime; IV—bright light and V—fluorescent (ethidium bromide-specific) images of the cell show it before (a) and after the generation of the 1st (b) and the 2nd (c) PNBs [85].

The generation and detection of tunable PNBs in living cells was studied in individual living A549 lung carcinoma cells. The cells were targeted with conjugates of 50 nm gold spheres to anti-epidermal growth factor receptor antibody C225 and were then exposed *in vitro* to a single pump laser pulse at a wavelength near the nanoparticle plasmon resonance peak (0.5 ns, 532 nm). Optical scattering of the pulsed probe beam (690 nm) by the gold NPs and by the PNBs in the cells was measured as image pixel amplitude (Figure 17-II). Also, the lifetime of the PNB was measured as the duration of a PNB-specific time response that was simultaneously obtained (Figure 17-III). We monitored the damage to the individual cells after their exposure to the laser pulse by fluorescent imaging of the uptake of Ethidium Bromide (which stains cells with a disrupted membrane) and blebbing (that is associated with damage of the cytoskeleton). Scattering by gold NPs accumulated by individual A549 cells after 30 minutes incubation at 37 °C (Figure 17a-II) was found to be quite low and its image amplitudes were close to the scattering image amplitudes associated with cellular organelles. We used the NP scattering image as a reference for quantifying the amplification of optical scattering by the PNBs. The first pump laser pulse was applied to individual cells at a fluence of 0.44 J/cm^2^ (above the bubble generation threshold), which induced a PNB within the cell which was detected with a probe laser image (Figure 17b-II). The lifetime of this PNB was relatively short, 25 ns, according to its time response (Figure 17b-III). This PNB amplified the scattering by 9.2 times relative to that of the gold NPs. After PNB generation, bright field (Figure 17b-IV) and fluorescent (Figure 17b-V) microscopy images of the cell showed no deviation from the pre-pulse conditions shown in Figure 17a-IV and Figure 17a-V, respectively. The absence of fluorescence and blebbing implied that the cell survived the laser pulse and the PNB. We detected only one PNB, despite the apparent fact that endocytosis assumes the internalization of many NPs. This can be explained by the threshold nature of the PNB: the fluence level was sufficient for the generation of the PNB only around the biggest clusters of NPs, while this fluence was below the PNB generation threshold for the smaller NP clusters or single NPs. This result demonstrates the high specificity of PNB generation compared to the specificity of nanoparticle imaging (Figure 17a-II). The sensitivity of PNB diagnosis *versus* NP diagnosis is clearly seen by comparing Figure 17a-II with Figure 17b-II: under identical imaging conditions the amplitude of NP scattering was so much lower than that for PNB scattering that it did not produce any detectible image.

Next, the second laser pulse was shortly applied to the same cell at the increased fluence of 3.2 J/cm^2^. The second PNB (Figure 17c-II) was much brighter, with its scattering amplitude being amplified 290-times relative to that of the NPs, and was also much longer (Figure 17c-III) than the first PNB. Within 30–60 s after PNB generation, the fluorescent image showed the penetration of the dye inside the cell (Figure 17c-V) and the bright field image showed the formation of blebbing bodies (Figure 17c-IV). These indicate the disruption of the cellular membrane and, possibly, of the cytoskeleton. This experiment demonstrates the ability to tune the intracellular PNB by varying the laser fluence from non-invasive imaging (with an almost 10-fold improvement in the optical scattering signal) to cell disruption.

### 3.3. In vivo Studies

**Figure 18.**
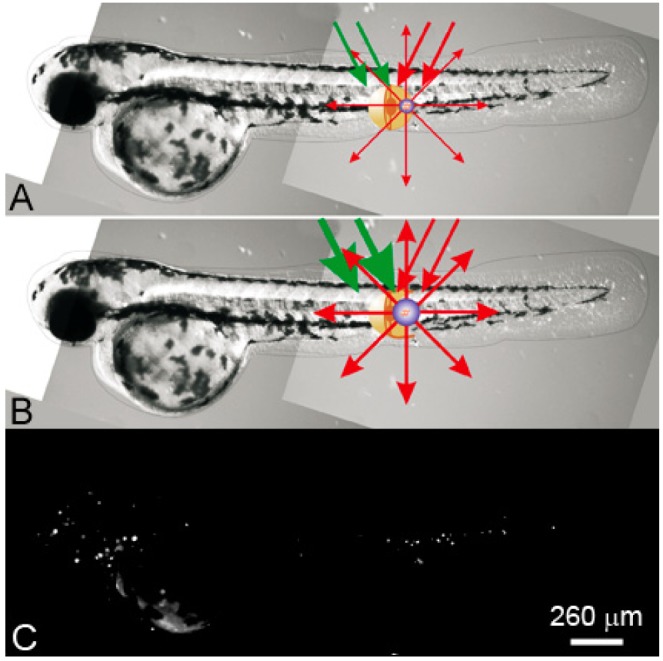
PNB theranostics *in vivo* includes the generation and detection of two sequential PNBs: (**a**) a small PNB is generated (with green pump laser pulse) in zebrafish in a specific cell and detected (with red probe laser pulse) thus sensing the cell; (**b**) the next, bigger PNB (generated with the second pulse of higher energy) destroys the cell, while optical scattering of the 2nd PNB guides the destruction; (**c**) fluorescent image of the embryo shows prostate labeled cancer cells scattered through its body [96].

Our *in vitro* and cell culture experiments show that PNBs are a potentially powerful theranostic agent. The successful clinical development of new materials and technologies requires their *in vivo* validation. Due to the large size of most experimental models and the variable optical qualities of different tissues, transitioning from *in vitro* methods to *in vivo* is challenging for many nanotechnologies and nanomaterials. To support the transition of PNB theranostics, we combined the properties of PNBs as cancer cell agents [85] with the discovered properties of a small optically transparent *in vivo* model, the zebrafish embryo, in particular, its ability to tolerate and support the remote and non-invasive generation and detection of PNBs [122]. In this work we tested the potential of PNB theranostics *in vivo* and we generated, tuned and detected PNBs in human prostate cancer xenografts transplanted into zebrafish embryo hosts (Figure 18a). Cultured metastatic human prostate cancer cells C4-2B were labeled with 60 nm gold nanoparticles conjugated with C225 anti-EGFR antibodies (EGF receptor is over-expressed by these tumors) and DiI fluorescent dye to provide a label for viability and lineage tracing after transplantation (Figure 18c). We found that single human prostate cancer cells can be detected and ablated under optical guidance *in vivo* by tunable PNBs in a single theranostic procedure.

We developed a novel *in vivo* model by using zebrafish. The zebrafish is a vertebrate organism that is relatively optically transparent, develops quite fast and is physiologically similar to humans. The zebrafish was already evaluated for analysis of the distribution and toxicity of plasmonic (gold) NPs [123-125] and also allowed efficient optical manipulations including laser microsurgery and sensing [126-130]. These findings allowed us to consider zebrafish embryos for plasmonic nanomedicine (optical scattering diagnosis, photothermal diagnosis and therapy, ultrasound and optical methods for drug delivery, cell manipulation and surgery). Furthermore, the zebrafish is a model for diverse cancers [131-136], which are promising targets for plasmonic therapies. Therefore, the zebrafish has an excellent potential for nanophotonic medicine.

**Figure 19.**
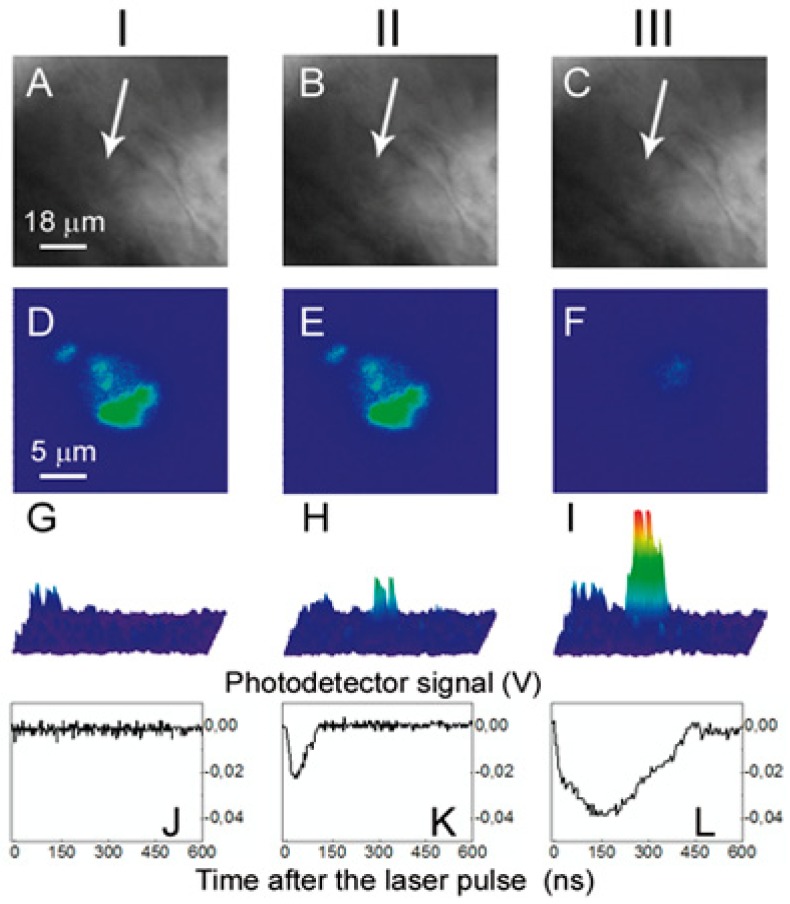
Images of the zebrafish embryo with prostate cancer cells incubated with gold NPs and DiI dye: I: before PNB, II: after the 1st PNB, III: after the 2nd PNB; (**a**-**c**) bright field images; (**d**–**f**) fluorescence of DiI ((**d**) before PNB, (**e**) after 1st PNB, (**f**) after 2nd PNB); (**g**–**i**) side scattering pulsed images of the cell (**g**), 1st PNB (**h**) and 2nd PNB (**i**); (**j**–**l**) corresponding time-responses obtained simultaneously with scattering images (**g**–**i**). Cancer cell shown with an arrow [96].

To test the applicability of PNB generation *in vivo*, we transplanted the fluorescent-labeled prostate cancer C4–2B cells into zebrafish embryos. Two days after transplantation, PC cells were distributed throughout the embryos with concentrations in the cardinal vessels and large numbers of single cells in the ventral tail veins (Figure 18d). Three conditions (NP-positive and NP-negative xenografts and ungrafted negative controls) were scanned with laser pulses under identical fluence. The embryos were imaged using bright field (Figure 18c, Figure 19a–19c), fluorescence (Figure 18d, Figure 19d–19f) and optical scattering (Figure 19g–19i). Using fluorescent imaging, we positioned the embryo so that each cancer cell matched the center of a laser beam (Figure 19d) because cancer cells cannot be distinguished from host tissue in the bright field (Figure 19a) and scattering images (Figure 19g). We scanned up to 20 cancer cells in each embryo. Each cancer cell was exposed to a single pump laser pulse #1 at a fluence of 125 mJ/cm^2^ (Figure 18a). We observed PNB-specific time response (Figure 19k) and bright spot-shaped scattering images (Figure 19h) only in the locations of the cancer cells. No PNBs were detected in non-fluorescent areas of the embryo including large heme rich blood vessels which can make PNBs under high fluence [122]. Thus we concluded that the PNBs were selectively generated only in cancer cells and not in surrounding normal host cells. The optical contrast of the first PNBs was found to be about one order of magnitude higher than that of the tissue scattering (Figure 19g and 19h, Table 3), and its lifetime was comparable to that obtained previously for cultured cells (Table 3, Figure 19k). The first PNB did not alter the bright field and fluorescent images (Figure 19b and 19e) but was bright enough to detect a single cancer cell in a tissue.

The same area of the embryo was then rapidly irradiated with a second pulse with a fluence of 175 mJ/cm^2^ (Figure 19b). The second PNBs were brighter and had higher contrast (Figure 19i, Table 3) with a five-fold longer lifetime than that of the first PNB (Table 3, Figure 19l). Within 20 s after the second PNB, we observed a loss of DiI fluorescence (Figure 19f) and concluded the cell was destroyed. However, we observed no changes in the bright field (Figure 19c) and scattering images of this area, indicating that the damage was limited to the target cell. Therefore, sequential PNB generation in a single cell demonstrated the three stages of theranostics: detection of a cancer cell with the first PNB, ablation of the detected cell with the second PNB, and real-time optical guidance of the cell destruction through the optical parameters of the second PNB.

The identical laser treatment of the control embryos containing C4–2B cells without NPs returned no PNBs at the fluence level of 175 mJ/cm^2^ in either cancer or embryonic cells (Table 4). Therefore, we concluded that the PNBs were generated specifically in NP-containing individual cancer cells. The PNB-treated embryos with cancer cells were observed for up to seven days after PNB generation and all of them survived the PNBs.

## 4. Plasmonic Nanobubbles and Other Cellular Probes

The concept of a PNB as a tunable cellular agent and of a cell theranostics with a remotely and dynamically tunable cellular probe significantly differs from laser- and NP- based methods, probes and technologies. Most current cellular probes were developed as particles, whose properties were pre-set at the fabrication stage, and which do not possess the stealth features of PNBs. Amongst all the aforementioned cellular probes, NPs demonstrated the widest functionality for imaging, delivery, and guidance [137-139], however, they cannot be tuned within a cell. The major drawback of most NPs (with the exception of gold nanoparticles of a relatively big size) is their lack of safety. Plasmonic gold NPs improve this problem [87], and demonstrate optical diagnostic [29,35,51,140] and therapeutic [29,32,51,63] potential. However, background scattering by cells and tissues often dominates the NP scattering signal, resulting in low sensitivity and specificity of NP-based diagnostic methods. Therapeutic NP technologies employ photothermal effects such as hyperthermia [29,51,63] and pressure or shock waves [141]. However, these are macro- rather than nano-scale effects, that cannot be localized and precisely controlled within single specific cells. Hyperthermia treatment requires a relatively long time (minutes), and, due to the inevitable thermal diffusion such treatment cannot be localized more precisely than within a millimeter range. Consequently gold NP hypothermia can damage healthy cells and tissues. The high cellular loads of nanoparticles (10^3–7^ NP/cell) required to support the effect, its low selectivity and tunability, together with the challenges of NP delivery, pose significant limitations to combining accurate diagnosis and targeted therapy at cell level.

Other agents such as gas filled, cavitation and vapor bubbles [36-49] provide diagnosis through acoustic detection, and therapy through heat and shock waves that result from bubble collapse, such as the HIFU (high intensity focused ultrasound) method. Despite clinical trials, such bubbles do not provide either tunability or cell level selectivity. Their generation requires a prolonged treatment time (up to six hours as compared to nanoseconds for PNBs), is significantly toxic (burns, pain and other adverse effects were reported), and depends upon additional ultrasound or magnetic resonance image guidance. The selectivity of such bubbles is in the millimeter range and is not appropriate for cell level. In contrast, PNBs were shown to act locally and without adverse thermal and shock wave effects [52,85,122]. The optical generation of thermal vapor bubbles in tissue was employed by laser surgery [75,142-146], and used the thermal ablative effects of laser radiation. Such ablative action cannot be localized to specific cells. Our proposed PNB theranostic technology fundamentally differs from current laser catheter systems (like those manufactured by Spectranetics: http://www.spectranetics.com/). Current laser surgery uses the target tissue as the heat source that generates macro-bubbles in the whole irradiated volume of millimeter scale, is associated with thermal impact [147,148] and cannot control such bubbles. Such macro-surgery fails to selectively ablate the target tumor and damages healthy tissues and even the fiber tip itself. PNB technology will use gold NPs as the local heat sources (instead of tissue) and will generate PNBs locally and at nanoscale (instead of macro-bubbles of millimeter size). In addition, no current laser technologies provide real-time cell level guidance and they require additional imaging equipment to guide the interventions. In contrast, monitoring the optical scattering signals of PNBs provides real-time guidance of their effect.

In addition to the above discussion, we would like to point out that none of the existing probes can provide true theranostic functions because the functional properties of such probes as nanoparticles, capsules and fluorescent markers cannot be tuned. In addition, their selectivity, sensitivity and safety are not sufficient for cell-level theranostics (Table 5).

The major limitation of the probes considered in Table 5 is their limited *in situ* dynamic tunability, although some can be tuned to a fixed function prior to their administration. Furthermore, most of these probes were designed for specific functions and cannot be tuned to support others. Despite widespread efforts, none of the above methods or probes are able to support imaging, delivery, therapy and guidance in one process with one agent at cell level and, consequently, they cannot support theranostics. Moreover, not all of the considered probes and agents provide cell level selectivity and specificity.

Apart from the methods and technologies considered above there is a relatively stand-alone group of methods based on extracorporeal flow analysis and treatment (flow cytometry, sorting and purging methods). Current approaches to high-throughput cytometry, sorting and purging employ CD34 antibodies conjugated to either magnetic beads or biotin to bind to CD34+ cells and then pass through selective columns to select CD34+ stem cells [151-156]. The limitations of such approaches include incomplete removal of contaminated cancer cells and the removal of important immune cells as mentioned above [151,155-157]. The efficacy of such purging is about a median of 3.1-log reduction of tumor cells with only about 50% of purging product being below the minimal detection limit of PCR assay [151-156]. Unlike the above technologies, PNB cell processing employs a dynamically tunable PNB probe that can detect and then ablate specific individual cells from amongst the bulk of other non-specific cells. Therefore, the PNB cell processing system will combine three steps in one rapid procedure: cell detection, follow up cell ablation and real-time guidance of the ablative process. None of the existing cytometry systems or technologies can support such a three-in-one operation.

While discussing the theranostic properties of various NPs and the methods for their activation and detection, we would like to point out another problem that is always associated with the research and validation of biomedical nanotechnologies: *in vivo* models. *In vivo* validation is an unavoidable stage during the translation of any new technology and material into their clinical applications. However, most of the existing animal models are associated with an additional challenge for all “nanotechnologies”: it is very difficult to trace and to monitor single NPs (or their minor amounts) in animals. This problem often results in excessive loading of NPs in order to exceed the detection limit, which, in turn, replaces the “nano” effects by “macro” effects. This is a common issue for detection of any NPs. To address it we developed an entirely novel *in vivo* model, a vertebrate animal, the zebrafish. We combined in zebrafish the optical excitation and detection of PNBs with the study of nanoparticles and a specific pathology, cancer. This improved accuracy in monitoring the theranostic effects of PNBs in the whole organism and with cell level sensitivity, without any damage to the host [96,122]. While being already successfully evaluated for the studies of nanoparticle biodistribution [123-125], laser manipulations and surgery [126-130], and cancer [131-136], such an animal was never before employed in the development of complex theranostic applications.

To conclude this discussion it is important to point out that PNBs, although they use the properties of both gold NPs and of the above discussed bubbles at nanoscale, are neither. To distinguish PNB from other biophotonic technologies that employ plasmonic (gold) NPs we compared three important parameters: dose of optical radiation, the NP load and selectivity of gold NP-based methods (Table 6).

As can be seen from the data in Table 6, PNBs require minimal optical doses, NP loads and can provide very good resolution. Superior performance of PNBs is provided by their localized nature, dual opto-mechanical function and by unique threshold mechanism of PNB generation with a single laser pulse. Another strategic advantage of PNBs, tunability of their function cannot be realized in any NP-based probes whose properties are pre-determined during fabrication and hence cannot be changed (tuned) in cells. Since a single PNB per cell is sufficient to support many functions, the PNB approach reduces the amount of NP load by 3-6 orders of magnitude. To summarize, the principal benefits of PNBs as cell probes are: (1) the dynamic tuning of their function in cells so as to support several processes with one agent: diagnosis, therapy and optical guidance of the therapy; (2) their single cell level of selectivity and specificity for diagnosis and treatment; (3) they are extremely safe since they are transient, on-demand phenomena and use safe gold nanoparticles with well-described targeting properties, but do not exist until activated with an optical pulse; (4) they can be applied to probe and treat pathological conditions at molecular, cellular and tissue levels.

## 5. Conclusions

Plasmonic nanobubbles (PNB), novel cellular probes, were discussed in terms of new medical application, theranostics. Given PNB flexibility and the use of non-toxic gold nanoparticles, we predict that this technology can be rapidly translated to the clinic for a variety of applications. PNBs can be developed in several directions: (1) Extra-corporeal cell processing systems for gene therapy and for monitoring and eliminating target cancer cells from blood and bone marrow; (2) Detection and elimination of residual cancer cell and microtumors on a thin surfaces (lung, head&neck and skin cancers) and in surgical bed (during the operation) and (3) Detection and ablation of residual cancer cells and metastases in deep tissues (in the way similar to brachytherapy of prostate and breast cancers). Tunable PNBs will influence also such fields as imaging, diagnosis, chemo- and gene therapy, cell level treatments, and micro-surgery. In addition, PNB technology is compatible with existing optical and laser instrumentation (microscopes, sorters, analyzers, devices for surgery and biopsy). This simplifies its commercialization and integration with existing research and clinical practices. Additional information and animated illustrations of PNB theranostics can be found on our website (http://lapotko.rice.edu/).

## Figures and Tables

**Table 1.** Comparison of the properties of plasmonic nanoparticles, vapor bubbles and plasmonic nanobubbles.

**Properties**	**Thermal**	**Mechanical**	**Optical**	**Threshold**	**Temporal Nature**
Nanoparticle	Impact to the environment with the radius being determined by duration of laser radiation	Acoustic wave	Permanent scattering	-	Steady
Nanobubble	Impact is confined inside PNB	Tunable and localized (to the NP location)	Tunable on demand scattering	+	Transient
Macro- and micro-bubble (photo- or sono-induced)	Impact to the environment after the collapse of the bubble	Cannot be tuned at nanoscale, localized to the energy source		+	Transient

**Table 2.** Gold NP load in a cell for the NP-based diagnosis and therapy with different methods.

	**PNB**	**Hyperthermia**	**Optical Scattering**	**Photoacoustic Imaging**
**Diagnosis**	100–1000 particles/cell	-	6500–50000 particles/cell	15000–13000*10^3^ particles/cell
**Therapy**	100–1000 particles/cell	5000–34000 particles/cell	-	-
**References**	[84,99]	[53,54]	[31,52]	[101]

**Table 3.** Parameters of PNBs and intracellular photothermal bubbles [85].

**Experimental Parameters**	**Process and Cell State**	**NP-treated Cells (PNB)**	**Intact cells (Cell Chromophore-Generated Photothermal Bubbles)**
Pump laser (532 nm, 0.5 ns) threshold fluence (J/cm^2^)	PNB generation	0.09 ± 0.03	2.72 ± 1.8
	Cell damage	1.0 ± 0.75	2.72 ± 1.8
PNB lifetime (ns)	Surviving cells	44 ± 17	n/a
	Damaged cells	213 ± 100	145 ± 50

**Table 4.** Plasmonic nanobubble parameters in three studied systems [96] (a.u.: arbitrary units).

		**Zebrafish Embryo**			**Cancer Cells*in vitro***		**Gold NPs in Water**	
Laser pulse fluence, mJ/cm^2^	**PNB Parameters**	**Cells with NPs and DiI**	**Cells with DiI**	**No Cancer Cells**	**NPs and DiI**	**DiI**	**NPs**	**NP Clusters**
	Generation threshold fluence, mJ/cm^2^	47	> 400	> 400	49	> 400	81	43
125 1st PNB	Probability, a.u.	1.0	0.0	0.0	1.0	0.04	0.95	1.0
	Lifetime, ns	62 ± 17	0	0	72 ± 15	0	45 ± 10	93 ± 30
	Optical contrast, a.u.	5.8 ± 3	1	1	15 ± 6	1	9 ± 3	28 ± 12
175 2nd PNB	Probability, a.u.	1.0	0.0	0.0	1.0	0.05	1.0	1.0
	Lifetime, ns	190 ± 52	0	0	230 ± 45	0	92 ± 16	210 ± 40
	Optical contrast, a.u.	19 ± 8	1	1	49 ± 23	1	27 ± 7	130 ± 30

**Table 5.** Comparison of the theranostic functions of the existing cellular probes and plasmonic nanobubbles (high—very efficient, low—not efficient).

**Probe**	**Function**				**Cell Level Selectivity and Specificity**	**Safety**
	**Diagnosis**	**Direct Therapy**	**Drug Delivery**	**Guidance**		
Fluorescent and luminescent probes [5-11]	high	low	low	high	high	low (chemical toxicity)
Micelles, polymers and liposomes [10,12-18]	low	low	high	low		depends upon release method
Nano-particles (general) [2,6-8,19-24]	high	high	high	low	low (for magnetic resonance and photo-acoustic imaging) high (for delivery and imaging)	low
Plasmonic nanoparticles [2,3,11,25-35]	high	high	high	low		high
Gas-filled and cavitation bubbles [36-49]	high	high	high	low	low	high
Plasmonic nanobubbles [59-61,85,86,96,99,100, 117,119,122,149, 150]	high	high	high	high	high	high

**Table 6.** Typical doses of optical radiation, NP loads per cell and spatial resolution/selectivity for gold NP-based technologies

**Technology**	**Optical Scattering Imaging**	**Photoacoustic Sensing**	**Photothermal Hypothermia**	**Delivery and Transfection**	**Laser Surgery**	**Plasmonic Nanobubbles**
Optical dose, (J/cm^2^)		0.002–0.012	20–26000	0.2–600	70-140	0.01-0.1
NP load, NP/cell	6500–50000	15000–13000 × 10^3^	5000–34000	32000–100000		100—1000
Spatial resolution (size of minimal target), (μm)	0.1	500	1000	10–100	10–100	0.1
References	[31,52]	[101-103]	[29,33,34,50, 51,53,54,63, 158-174]	[104-107]	[175, 176]	[56,57, 59-61,84,85, 96,99]
